# The neoadjuvant paradigm reinvigorated: a review of pre-surgical immunotherapy in HNSCC

**DOI:** 10.1186/s41199-020-00052-8

**Published:** 2020-03-16

**Authors:** Margaret Stafford, John Kaczmar

**Affiliations:** Division of Hematology and Oncology, Medical University of South Carolina, 39 Sabin Street, MSC 635, Charleston, SC 29425 USA

**Keywords:** Head and neck cancer, Squamous cell carcinoma, Neoadjuvant, Induction therapy, Immunotherapy, Immune checkpoint inhibitor, Immune profile

## Abstract

**Background:**

There remains up to a 50% recurrence rate in advanced p16- head and neck squamous cell carcinoma with current standard of care treatment. In an attempt to improve survival, multiple trials administering induction or neoadjuvant chemotherapy have been conducted but none demonstrated improved overall survival. The established efficacy of immune checkpoint inhibitors in the recurrent and metastatic setting has produced widespread interest in their neoadjuvant use.

**Purpose:**

To survey the landscape of active neoadjuvant immunotherapy trials in head and neck squamous cell carcinoma and summarize and synthesize currently available outcomes from these trials.

**Conclusions:**

Neoadjuvant immunotherapy has proven safe and well tolerated in head and neck squamous cell carcinoma with encouraging efficacy results, including relatively high rates of pathologic response. Ongoing studies offer an opportunity to study immune responses in vivo. PD-L1 positivity, high tumor mutational burden and infiltration of NK cells, CD8, CD26 and Tim3 positive lymphocytes at time of surgery have been correlated with pathologic responses. We await updated reports of disease free survival and overall survival data and results of ongoing phase III studies utilizing neoadjuvant immunotherapy to determine if this treatment paradigm will have a place in the standard of care treatment in head and neck squamous cell carcinoma.

## Background

Current standard of care treatment for patients with locoregionally advanced head and neck squamous cell carcinoma (HNSCC) is either definitive chemoradiotherapy (CRT) or surgical resection followed by adjuvant radiation with the addition of radiosensitizing chemotherapy when high risk features are noted on final pathology. Even with this aggressive approach, there remains up to a 50% recurrence rate in advanced Human papillomavirus (HPV) unrelated, p16- HNSCC. P16+ HNSCC has a far more favorable response to therapy with significantly lower rates of recurrence reflected in the development of updated staging reflected in the AJCC 8th edition [1]. Despite this improved prognosis, distant recurrences remain a problem in HPV initiated HNSCC and appear to occur later [2].

## Induction chemotherapy in non-surgical management

With a goal toward improving survival outcomes in HNSCC there has historically been interest in the application of induction chemotherapy. Results of 2 major well designed trials (PARADIGM and DeCIDE) failed to demonstrate improved overall survival (OS) with the addition of induction chemotherapy to CRT [3, 4]. Concerns about toxicity were raised by the the DeCIDE trial, which demonstrated a significant increase in serious adverse events in the induction therapy arm (47% vs 28% *p* = 0.002). The most common grade 3 and 4 toxicities during induction were febrile neutropenia (11%) and mucositis (9%). Impact of induction chemotherapy on absolute neutrophil count persisted through CRT with grade 3 neutropenia in 14% of the induction arm and 3.8% in the control arm (*p* = 0.039). These studies were both unfortunately underpowered due to failure to meet target enrollment.

Hitt et al. enrolled 439 patients with locally advanced, unresectable, HNSCC and randomized to induction TPF (docetaxel, cisplatin, 5-FU) followed by CRT, PF (cisplatin and 5-FU) followed by CRT, or CRT alone and found no difference in progression free survival (PFS) or time to treatment failure [5]. Again, there was substantially increased toxicity in the induction chemotherapy arms. Grade 3–4 febrile neutropenia occurred in 17% of patients in the TPF arm and 1.9% of patients in the PF arm. Rates of grade 3–4 odynophagia and stomatitis were higher during CRT in both the induction chemotherapy arms than in the CRT alone arm. Thirteen deaths due to study treatment toxicity were reported: seven in the TPF and CRT arm (4.5%), four in the PF and CRT arm (2.6%), and two in the CRT alone arm (1.6%). These were mainly by febrile neutropenia before G-CSF use was implemented. A high quality meta-analysis failed to define a clear benefit of induction chemotherapy and supported CRT as the primary non-surgical treatment modality [6, 7].

More recently, GORTEC 2007–02 showed no improvement in PFS or OS with induction chemotherapy followed by cetuximab radiotherapy compared to chemoradiotherapy, though there was a reduction in the rate of distant metastases (DM) in the induction chemotherapy arm [8]. p16 status was stratified (*n* = 26, 31% in CRT arm, *n* = 19, 21% in TPF and cetuximab RT arm), but results were consistent with the overall population. A significant increase in grade 3 and 4 fever, neutropenia, and febrile neutropenia was also observed. Despite the recommended prophylactic use of lenograstim and the systemic use of ciprofloxacin, 12 patients died during or in the 30 days after TPF (6.6%). All deaths, except one, were considered to be related to TPF. The decrease in DM noted in the GORTEC 2007–02 study and the trend to lower DM noted in the DeCIDE trial suggests there may yet be a subset of patients who could benefit from induction chemotherapy, but this group remains inadequately defined.

Most induction chemotherapy studies were designed prior to the widespread incorporation of p16 testing and stratification into prospective studies of HNSCC. Given the improved prognosis related to HPV initiated HNSCC, interest in induction therapy has primarily been related to the idea of chemoselection – with a goal toward treatment de-escalation [9]. ECOG 1308 demonstrated the feasibility of such a schema, but whether induction chemotherapy need be part of future de-escalation regimens remains controversial and its use of cetuximab, which has been shown to be an inferior radiosensitizer to cisplatin, make this an unlikely platform to move forward [10, 11].

Induction chemotherapy may still be considered in select clinical situations after multi-disciplinary discussion, but its routine use is not supported by the available data, except as a treatment option in larynx preservation [12]. When induction chemotherapy is administered, TPF is the preferred regimen [13, 14]. The potential of increased toxicity interfering with receipt of chemoradiation remains a challenge [4, 15].

## Neoadjuvant chemotherapy

As with induction chemotherapy, there is a lack of evidence to support the routine administration of chemotherapy prior to surgery (neoadjuvant chemotherapy) in patients with resectable HNSCC. A study of patients with stage T2-T4, N0-N2 previously untreated SCC of the oral cavity (OSCC) failed to show a significant difference in locoregional relapse or survival with the addition of neoadjuvant PF [16]. Of interest, patients with a pathologic complete response (pCR) had a higher probability of survival than those without (10-year OS: 76.2% versus 41.3%, *p* = 0.0004). A phase III trial demonstrated an excellent response rate of 80.6%, but did not show a survival benefit with neoadjuvant TPF in stage III or IVA OSCC in patients undergoing radical surgery and postoperative radiation therapy [17]. Clinical response was assessed by Response Evaluation Criteria in Solid Tumors (RECIST) version 1.0 at baseline and 2 weeks after cycle 2 of neoadjuvant chemotherapy. Patients who achieved a clinical response or favorable pathologic response (< 10% viable tumor cells) had improved OS and locoregional and distant control. A small meta-analysis did not show any improvement in clinical outcomes with neoadjuvant chemotherapy in patients with advanced OSCC [18]. Subgroup analysis of cN2 patients demonstrated a significant benefit in overall survival in favor of the neoadjuvant chemotherapy group, however this result must be interpreted with caution given the small number of patients (*n* = 52, 20.3%) with cN2 HNSCC in the study and the small number of events related to the assessed clinical outcomes.

The induction/neoadjuvant treatment paradigm in HNSCC remains alluring, both as a part of treatment intensification in HPV- HNSCC and a potential driver of deintensification in HPV+ HNSCC. The vast majority of ongoing trials of neoadjuvant therapy in HNSCC now are designed with immunotherapy as the foundation. This foundation is built on the success of immunotherapy in the recurrent and metastatic setting.

## Success of immunotherapy in recurrent/metastatic (R/M) disease

Immunotherapy has a well-established role in recurrent and metastatic HNSCC as a second line agent following platinum-based chemotherapy. Checkmate 141 demonstrated superiority of anti-programmed death 1 (PD-1) monoclonal antibody nivolumab versus standard single agent therapy of the investigator’s choice in recurrent or metastatic HNSCC refractory to platinum chemotherapy [19]. Median OS in the nivolumab arm was 7.5 months versus 5.1 months in the control group. Subsequently, KEYNOTE-040 yielded similar results utilizing pembrolizumab resulting in median OS in the intention-to-treat population of 8.4 months with pembrolizumab and 6.9 months with standard of care treatment [20].

More recently, data from KEYNOTE-048 support first line use of pembrolizumab with or without chemotherapy as first line therapy in recurrent/metastatic head and neck cancer [21, 22]. In the trial, pembrolizumab or pembrolizumab, platinum chemotherapy, and 5-fluorouracil were compared with the EXTREME regimen. There was a significant improvement in OS with pembrolizumab monotherapy over EXTREME in the programmed death-ligand 1 (PD-L1) combined positive score (CPS) ≥ 20 and PD-L1 ≥ 1 populations. All cause grade 3–5 adverse events were lowest in the pembrolizumab monotherapy group at 54.7, 85.1% in the pembrolizumab plus chemotherapy group, and 83.3% for the EXTREME regimen group. While serious toxicities from immune checkpoint inhibitors can occur, immunotherapy has proven to be better tolerated than chemotherapy overall. Available data has shown improved quality of life with immunotherapy compared to chemotherapy in the recurrent/metastatic setting [11, 23].

The established efficacy of immune checkpoint inhibitors in the recurrent and metastatic setting has produced widespread interest in their neoadjuvant use. Neoadjuvant immunotherapy in HNSCC is appealing for a host of reasons. Incorporation of immunotherapy in the curative setting might reduce the risk of local recurrence and distant metastasis thereby improving overall survival. Opportunities to potentially de-escalate adjuvant therapy or the potential to reduce the morbidity and necessary extent of surgical resection are intriguing as well. Investigational use of neoadjuvant immunotherapy also offers an ideal platform to perform correlative studies to assess whether improved predictors of response to immunotherapy might be found. Finally, neoadjuvant immunotherapy is expected to be more easily tolerated than TPF and as a result would be unlikely to limit dose intensity of adjuvant cisplatin based chemoradiation when indicated.

The purpose of this review is to survey the field of active neoadjuvant immunotherapy trials in HNSCC and summarize currently available outcomes from these trials. Most of these trials are investigating efficacy and safety of immune checkpoint inhibitors, but cytokine therapy and vaccine studies are also ongoing (Table 1).

**Table 1:** Active and recently posted neoadjuvant immunotherapy trials

Trial number	Phase	Neoadjuvant therapy
NCT03700905^a^	III	nivolumab
NCT03765918^a^	III	pembrolizumab
NCT02002182	II	ADXS11–001 (ADXS-HPV) vaccine
NCT02296684^ar^	II	pembrolizumab
NCT02488759^r^	I/II	nivolumab
NCT02609386^a^	II	IRX-2 plus cyclophosphamide, then indomethacin, zinc-containing vitamins and omeprazole
NCT02641093^ar^	II	pembrolizumab
NCT03003637^r^	I/II	ipilimumab and nivolumab
NCT03021993^r^	II	nivolumab
NCT03107182^a^	II	nab-paclitaxel, carboplatin, nivolumab
NCT03174275^a^	II	carboplatin, nab-paclitaxel, durvalumab
NCT03247712^a^	I/II	nivolumab
NCT03342911	II	carboplatin, paclitaxel, nivolumab
NCT03708224^a^	II	atezolizumab +/− emactuzumab
NCT03721757^a^	II	nivolumab
NCT03737968^a^	II	durvalumab +/− tremelimumab
NCT03916627^a^	II	cemiplimab
NCT03944915	II	carboplatin, paclitaxel, and nivolumab
NCT03129061	I	carboplatin, nab-paclitaxel, durvalumab
NCT03144778^r^	I	durvalumab +/− tremelimumab
NCT03635164^a^	I/Ib	RT + durvalumab
NCT03843515	I	nivolumab

a = adjuvant immunotherapy, r = results reported, *RT* radiation therapy

## Neoadjuvant immunotherapy trial results

Prior to the immune checkpoint inhibitor era, Stefani et al. reported improved OS and disease free survival utilizing neoadjuvant perilymphatic interleukin 2 (IL-2) in patients with resectable squamous cell carcinoma of the oral cavity and oropharynx [24]. Patients with a resectable T2-T4,N0-N3,M0 SCC of the oral cavity and oropharynx were assigned randomly to receive surgery and radiotherapy or to receive IL-2, surgery, and radiotherapy. rIL-2 was injected around the ipsilateral cervical lymph node chain daily for 10 days before surgery. After surgery, contralateral 5-day rIL-2 courses were administered monthly for 1 year. There were some late recurrences after 5 years in the rIL-2 group, suggesting that some of the OS benefit may derive from delayed recurrence related to rIL-2 therapy. This treatment tactic was never integrated into mainstream practice but serves as an interesting historical example that lends some credence to a neoadjuvant, and potentially also adjuvant, immunotherapy strategy.

## Uppaluri et al.

Findings from NCT02296684, a trial of neoadjuvant pembrolizumab in surgically resectable stage III and IV HPV- head and neck cancer were presented at the American Society of Clinical Oncology (ASCO) 2017 annual meeting [25]. To our knowledge, this is the first described experience with neoadjuvant immunotherapy in HNSCC. All 25 enrolled patients were given 1 dose of pembrolizumab 2 to 3 weeks prior to definitive surgical resection. Patients with high-risk features received radiation and cisplatin with adjuvant pembrolizumab while those without high-risk features received standard of care adjuvant therapy.

Results reported thus far are favorable. There were no surgical delays or unexpected adverse events (specific observed immunotherapy-related adverse events have not yet been reported). There were no reported hyperprogressions or pseudoprogressions. The majority of patients in the study were locally advanced with stage IV disease. Despite this, there were no locoregional recurrences or distant metastases at the time of 1 year follow up from surgery in the first 14 patients for whom 1 year follow up data was available. 43% of patients had a pathologic treatment response (> 10% tumor necrosis and/or giant cell/histiocytic reaction to keratinous debris) at the time of surgery following a single dose of pembrolizumab. Pathologic treatment effect (TE) in ≥70% of the resected tumor or lymph node tissue area occurred in 6/21 pts. (29%). Pre-treatment tumor cell PD-L1 staining correlated with the percent treatment effect. Additionally, 48% of patients had clinical to pathological treatment downstaging.

One patient whose contralateral neck dissection was performed weeks after his initial surgery demonstrated an additional treatment response in the contralateral lymph nodes. This could represent a delayed treatment effect from his initial pembrolizumab dose. It is also possible that surgery itself promoted a further immune-mediated anti-tumor response.

## Wise-Draper et al.

Initial results from a multisite phase II trial investigating neoadjuvant and adjuvant pembrolizumab combined with adjuvant radiation plus or minus cisplatin (NCT02641093) were presented at the ASCO 2018 annual meeting [26]. Clinically high-risk patients were enrolled (T3–4 and/or 2 or more positive lymph nodes). HPV+ oropharyngeal cancer patients were excluded. All patients received a single dose of neoadjuvant pembrolizumab 1–3 weeks before surgery. Based on risk stratification at the time of surgery, patients received either radiation plus pembrolizumab or weekly cisplatin and radiation plus pembrolizumab in the adjuvant setting. The primary objectives were to determine the frequency and severity of side effects of pembrolizumab in combination with radiation and CRT in the adjuvant setting and 1- and 3-year disease free survival in both groups.

In the completed safety lead in including 16 patients no toxicities occurred resulting in delayed surgery, radiation, or chemotherapy. Enrollment was ongoing at the time of data presentation. In the first 34 patients, grade 3 pembrolizumab-associated toxicities included 1 event each of autoimmune colitis, duodenal hemorrhage, mucositis, nausea, vomiting, and syncope. There were no grade 4 pembrolizumab-associated toxicities. 14/27 (52%) patient’s tumors demonstrated a pathological response, defined as ≥10% tumor necrosis and/or histiocytic reaction to keratinaceous debris or ≥ 10% decrease in viable tumor. One patient had a complete pathologic response after one dose of pembrolizumab. Many tumors (14/34, 41%) were down-staged based on clinical to pathologic stage comparison. There were no reported hyperprogressions related to immunotherapy. Clinical response was assessed at 3 months post radiation or at the time of relapse with imaging and exam. 5 patients developed progressive disease or recurrence following surgery and RT or CRT with pembrolizumab. None of the patients who developed progressive or recurrent disease post-operatively by clinical response assessment had a pathologic response at the time of initial surgery. Pathological response was associated with robust immune cell infiltration, particularly CD8 positive T cells at time of surgery and PD-L1 and PD-L2 positivity at both pre-treatment biopsy and time of surgery.

A randomized phase III trial utilizing a slightly modified treatment protocol in the experimental arm is now underway (NCT03765918).

## Horton et al.

Results for the first stage of a planned two-stage, single arm, phase II clinical trial utilizing neoadjuvant nivolumab in oral cavity squamous cell carcinoma (OCSCC) (NCT03021993) were recently presented [27]. Nine patients with stage II-IVa OCSCC received 3–4 biweekly doses of 3 mg/kg nivolumab followed by definitive surgical resection with curative intent. Upon enrollment, patients received nivolumab 3 mg/kg intravenously (IV) every 2 weeks for a total of three doses prior to interval radiologic evaluation between study days 28–35. In the event of disease progression, patients were taken for definitive surgical resection between days 36–42. Patients with radiographically stable disease or response received a fourth dose of nivolumab on day 43 +/− 1 followed by definitive surgical resection on day 50–56.

The primary endpoint was response to treatment. Response to treatment was defined as a pathologic complete response or pathologic partial response (30% reduction in tumor size in the surgical specimen compared to pre-treatment radiologic imaging). Secondary endpoints included safety, feasibility, and memory phenotype and surface marker expression of ex vivo expanded tumor infiltrating lymphocytes (TILs).

There was a 44% (4/9) objective overall response rate. Each of the 4 patients with response had a partial response. There were no pathologic complete responses (CRs). 1 additional patient had stable disease. The remaining 4 patients progressed through treatment. One patient’s tumor was felt to experience hyperprogression, having nearly doubled in size, but was still able to undergo definitive surgical resection. Anecdotally, those patients with responses had clinical findings of reduced cancer associated pain, trismus, or reduced extent of the primary tumor at 2 weeks after the first treatment. At a median follow up of 10 months, 3 patients had recurred with 1 death due to recurrence. The remaining 6 were recurrence free. There were no delays in surgical treatment. Negative margins were achieved in all patients. There were no grade 3 or 4 adverse events (specific adverse events are not yet reported). There were no treatment interruptions.

Clinical response correlated with pathologic response. All patients who had a pathologic response had also demonstrated response on their pre-surgery interval CT scan. This is in line with observations in the recurrent and metastatic setting. While pseudo-progression is a concern in other cancer types with immune checkpoint inhibitors, it appears to be quite uncommon in head and neck cancers [28].

An increased lymphocytic infiltrate was observed in pathology from responders compared to non-responders. The authors were able to demonstrate an immune profile of TILs in surgical specimens [29]. There was a significant correlation between response to treatment and increased CD-26 and Tim3 expression. In non-responders there was an increase in effector memory T cell infiltration and CD4+ T cells. PD-L1 expression did not significantly correlate with response. There was a positive linear association of post-nivolumab serum neutrophil-to-lymphocyte ratio (NLR) with change in tumor size. Tumor growth correlated to an increased neutrophil-to-lymphocyte ratio. Absolute neutrophil count did not correlate [30].

## CheckMate 358

Preliminary results from the head and neck cohort of Checkmate 358 were presented at the 2017 ESMO Conference. CheckMate 358 (NCT02488759) is exploring the safety and feasibility of neoadjuvant nivolumab in patients with resectable HPV+ or HPV- HNSCC [31]. Patients had T1 or greater primary lesions and N1 or greater nodal disease. Patients received 2 doses of nivolumab in the neoadjuvant setting. Twenty-nine patients were enrolled, 12 with HPV+ and 17 with HPV- tumors. Treatment related adverse events did not result in any protocol-defined surgery delays. Grade 3–4 treatment-related adverse events occurred in 2 (16.7%) patients with HPV+ tumors (lipase increased and glossodynia) and 2 (11.8%) pts. with HPV- tumors (lipase increased). No new safety signals were reported. As of database lock, pre-surgery tumor reduction by CT scan was observed in 11 of 23 (48%) evaluable pts. (5/10 HPV+ and 6/13 HPV-). Three patients had tumor reduction ≥40% with the largest reduction being 75%. Pathologic data has not been reported.

## Ciao

Findings of the Checkpoint Inhibitors Assessment in Oropharynx Carcinoma Study (CIAO) (NCT03144778) were presented at ASCO 2019 [32]. Twenty-nine patients with stage II-IVA oropharyngeal SCC were randomized to receive neoadjuvant durvalumab (anti-PD-L1) or durvalumab plus tremelimumab (a CTLA-4 antibody). Unlike the previously described trials, this study was open to patients with recurrent disease as well as treatment-naïve patients. For patients to be eligible with recurrent disease, they must have a loco-regional recurrence from an OPSCC primary and the time to recurrence must be at least 6 months after completion of initial curative intent treatment (surgery or RT +/− chemotherapy or cetuximab). Both HPV+ and HPV- patients were eligible. The primary objective was to compare CD8+ TIL at the time of surgery in the two arms with the hypothesis being that there would be an increased proportion of CD8+ TIL in the combined durvalumab plus tremelimumab group resulting in an amplified anti-tumor response. Patients received durvalumab or durvalumab plus tremelimumab on days 1 and 29 followed by surgery between days 52–72 and adjuvant RT vs CRT based on standard of care pathologic risk stratification. Radiographic tumor assessment was performed at 8 weeks for objective response determination by RECIST 1.1. The enrolled population was heterogenous with the majority of patients being stage III (*n* = 6, 21%) or stage IVA (*n* = 13, 45%). Nine patients (31%) had locally recurrent disease were included in the study. The majority of patients were p16 positive (*n* = 25, 86%). 96% were male and 76% were non-smokers.

Durvalumab plus tremelimumab did not increase CD8+ TIL or response compared to durvalumab alone. The majority of patients (26/29, 90%) received the 2 planned immunotherapy cycles. Two patients progressed during treatment. These two patients, along with a third who had stable disease and was considered borderline resectable, were switched to treatment with neoadjuvant chemotherapy and, therefore, were excluded from the pathologic response and primary response assessments. All patients underwent the planned surgery, though some surgeries (3/28, 11%) did take place outside the planned window. Most patients did not receive adjuvant radiation therapy (57%). There was no difference in the toxicity profile between the durvalumab group and durvalumab plus tremelimumab group. Grade 2 and 3 toxicities included increased AST/ALT, increased lipase, and hypothyroidism. 4 (13.7%) of patients experienced grade 3 toxicities (increased AST/ALT, increased lipase, and diarrhea). Mean average quality-of-life scores using the MDASI-HN 28 item multi-symptom inventory were low at baseline and throughout treatment in both arms.

Two patients had a pathologic CR at the primary tumor (1 treated with durvalumab and 1 treated with durvalumab plus tremelimumab). 32% had a major pathological response, defined as less than or equal to 10% viable tumor cells in the primary or nodal disease site. The overall response rate by RECIST was 43% in both groups, including a 50% response in patients with recurrent disease. Within the primary tumor there was excellent correlation between response defined by imaging and pathologic response. Imaging was not as strongly predictive of nodal response. No patients had recurred at the overall median follow up of 10.5 months.

## IMCISION

Results from the phase 1b portion of immunomodulation by the combination of ipilimumab and nivolumab neoadjuvant to (salvage) surgery in advanced or recurrent head and neck carcinoma, IMCISION (NCT03003637) were also presented at ASCO 2019 [33]. Twelve patients with advanced (T3–4,N0–3, M0) HNSCC eligible for curative-intent surgical resection were enrolled. Like CIAO, this study included both therapy-naïve patients and patients with recurrent disease. Though HPV+ patients were included per study protocol, only HPV- patients had been enrolled at the time of reporting. 7/12 (58%) of patients had ACE-27 grade 2–3 comorbidities. Patients were assigned to arms utilizing a 3 + 3 design. In Arm A, 6 patients (5/6 undergoing salvage surgery) received nivolumab 240 mg flat dose weeks 1 and 3. In Arm B, 6 patients (2/6 undergoing salvage surgery) received ipilimumab week 1 only and nivolumab weeks 1 and 3 (ipi/nivo). Surgery was completed week 5 for all patients followed by standard of care adjuvant therapy.

There were no surgical delays. One patient with recurrent disease after definitive radiotherapy had a greater than 50% increase from baseline local tumor size. In total, grade 1–4 immune-related toxicity occurred in 4/6 (67%) patients in arm A and 3/6 (50%) in arm B. One patient developed grade 3–4 colitis in the nivolumab arm. In the ipi/nivo arm there were 2 grade 3–4 toxicities, 1 incident of hepatitis and 1 of colitis. No unexpected wound healing problems were observed.

1/6 (17%) patients in the nivolumab arm had a pathologic response (this was a near complete response [pCR], > 90% response). 3/6 (50%) in the combined ipi/nivo arm had a pathologic response (> 50% pathologic response) with 2 near pCRs. No patients with a near pCR had a recurrence at latest follow up (median 15 months). It is important to note the increased proportion of patients with recurrent disease undergoing salvage surgery in the nivolumab arm.

Patients with a near pCR (*n* = 3) had 1–50% PD-L1 (*n* = 2) and/or high tumor mutational burden (TMB) (*n* = 2). TMB was a significant predictor for pathological response (*p* < 0.05). Increased B7-H3 gene expression was detected in non-responders before treatment. Increased NK cell gene expression was detected in responders post treatment. Intra-tumoral NK cell influx was confirmed by immunohistochemistry.

## Discussion

In addition to these studies highlighted, numerous other studies (Table 1) will report out data in the coming months and years, expanding our understanding of neoadjuvant immunotherapy in HNSCC. Outcomes highlighted so far demonstrate high rates of pathologic response after as few as one dose of checkpoint inhibition. Rates of pathologic response in a heterogeneous population of patients with HNSCC ranged from 17 to 52%. A significant proportion of patients had pathologic downstaging, which in some patients likely allowed for de-intensified adjuvant therapy with RT rather than CRT. More data and longer term follow-up is needed to determine if de-escalation in patients with response to neoadjuvant immunotherapy is safe and cannot be recommended at this time. Encouragingly, few patients progressed through immunotherapy and those who did were all able to undergo definitive surgery. Only the CIAO study had any reported delays in surgery and that study also included patients with recurrent disease. In contrast to neoaduvant and induction chemotherapy trials, there have been no deaths reported at this time related to neoadjuvant immunotherapy. Neoadjuvant immunotherapy in these pre-surgical populations has, to date, proven safe and observed immunotherapy-related toxicities are in line with those observed in larger phase III trials.

Notably, response rates, though generally impressive appear lower than those seen with TPF chemotherapy, but significantly greater than those seen with single agent immunotherapy in the recurrent and metastatic setting [14]. Response rates have proven a poor marker of the efficacy of immunotherapy and the true measure of the success or failure of neoadjuvant immunotherapy must be in the hard end points of disease free survival (DFS) and OS. Tantalizingly, preliminary survival and recurrence rates are favorable, but longer term follow-up and more patient data is needed before any firm conclusions can be made.

Predictors of response to neoadjuvant therapy in HNSCC are lacking, as are those of response to immunotherapy writ large. Many of these studies have built in robust pre-specified correlative analyses to potentially identify signatures that predict response or lack thereof to immunotherapy. While much data remains preliminary, there are early signals toward association of response to therapy with PD-L1 expression and high pre-treatment TMB [23]. Of interest, association of major pathological response with increased TMB was also seen following neoadjuvant immunotherapy in non-small cell lung-cancer [34]. Additionally, at the time of surgery in HNSCC, increased infiltration of CD8, CD-26, and Tim3 positive TIL were observed in responders, while an increase in effector memory T cells was noted in non-responders [20]. Increased NK cell infiltrate has also been observed in responders at time of surgery [23]. Further development of these immune signatures could serve as a powerful prognostic guide, especially should long term follow-up demonstrate responders have more favorable OS and DFS. Utilizing and defining tumor markers to guide therapy will remain an important ongoing area of investigation.

The optimal treatment for those patients who do recur after neoadjuvant immunotherapy will be an increasingly important question to study. It is unclear if recurrent tumors would retain responsiveness to PD-1/PD-L1 antibodies or if those that did not respond in the neoadjuvant setting might prove more responsive in R/M setting. In the metastatic setting, there are multiple drugs undergoing investigation seeking to recapture therapeutic benefit with immunotherapy in patients who have previously responded [35].

## Conclusion

Neoadjuvant immunotherapy has proven safe and well tolerated in HNSCC with encouraging efficacy results, to date. We await further reports of DFS and OS data and results of ongoing phase III studies utilizing neoadjuvant immunotherapy to help determine if this treatment paradigm will have a place in the standard of care treatment in HNSCC. Should a role for neoadjuvant immunotherapy be established there will still be many questions to answer including: duration of neoadjuvant treatment, whether there is a role for the addition of dual checkpoint inhibition or chemo-immunotherapy, and whether the addition of adjuvant immunotherapy provides any additional benefit. Unfortunately, many ongoing neoadjuvant studies feature adjuvant immunotherapy, which will make it more difficult to determine the optimal sequencing of immunotherapy in HNSCC. In an increasingly cost-conscious era of medicine, the optimal sequencing and duration of immunotherapy in the curative setting will be an exceedingly important question to answer. Finally, the promise of defining predictors of response to immunotherapy is increasingly real and may help select patients who would benefit from neoadjuvant immunotherapy and those who should instead proceed to current standard of care treatments. Immunotherapy has and is continuing to change the treatment landscape of recurrent and metastatic disease and now promises to change the face of curative intent therapy as well.

## Data Availability

Data sharing not applicable to this article as no datasets were generated or analyzed during the current study.

## Abbreviations

ACE-27: Adult Comorbidity Evaluation-27
AJCC: American Joint Committee on Cancer
ALT: Alanine Aminotransferase
ASCO: American Society of Clinical Oncology
AST: Aspartate Aminotransferase
CIAO: Checkpoint Inhibitors Assessment in Oropharynx Carcinoma Study
CR: Complete response
CRT: Chemoradiotherapy
CT: Computed tomography
DFS: Disease free survival
HNSCC: Head and neck squamous cell carcinoma
HPV: Human papillomavirus
IL-2: Interleukin 2
IV: Intravenously
MDASI-HN: M. D. Anderson Symptom Inventory- Head and Neck
NK: Natural killer
NLR: Neutrophil-to-lymphocyte ratio
OS: Overall survival
OSCC: Oral cavity squamous cell carcinoma
pCR: Pathologic complete response
PD-1: Programmed death 1
PD-L1: Programmed death-ligand 1
PD-L2: Programmed death-ligand 2
PF: Cisplatin and 5-fluorouracil
RECIST: Response evaluation criteria in solid tumors
TE: Treatment effect
TIL: Tumor infiltrating lymphocytes
TPF: Docetaxel (Taxotere), cisplatin, and 5-fluorouracil
